# Surgical Management of Patients with Parotid Involvement from Non-Melanoma Skin Cancer of the Head and Neck

**DOI:** 10.3390/jpm14060631

**Published:** 2024-06-13

**Authors:** Filippo Carta, Simone Corrias, Melania Tatti, Valeria Marrosu, Mauro Bontempi, Cinzia Mariani, Clara Gerosa, Caterina Ferreli, Matteo Atzeni, Filippo Boriani, Andrea Figus, Roberto Puxeddu

**Affiliations:** 1 Unit of Otorhinolaryngology, Department of Surgery, Azienda Ospedaliero-Universitaria di Cagliari, University of Cagliari, 09100 Cagliari, Italy; s.corrias12@studenti.unica.it (S.C.); m.tatti@aoucagliari.it (M.T.); v.marrosu@aoucagliari.it (V.M.); m.bontempi@aoucagliari.it (M.B.); cinzia.mariani@unica.it (C.M.); puxeddu@unica.it (R.P.); 2 Unit of Pathology, Department of Medical Sciences and Public Health, Azienda Ospedaliero-Universitaria di Cagliari, University of Cagliari, 09100 Cagliari, Italy; clara.gerosa@unica.it; 3 Unit of Dermatology, Department of Medical Sciences, Azienda Ospedaliero-Universitaria di Cagliari, University of Cagliari, 09100 Cagliari, Italy; ferreli@unica.it; 4 Unit of Plastic Surgery, Department of Surgery, Azienda Ospedaliero-Universitaria di Cagliari, University of Cagliari, 09100 Cagliari, Italy; m.atzeni@aoucagliari.it (M.A.); filippo.boriani@unica.it (F.B.); andreafigus@unica.it (A.F.); 5 Unit of Otorhinolaryngology, Department of Surgery, King’s College Hospital London-Dubai, Dubai P.O. Box 340901, United Arab Emirates

**Keywords:** parotidectomy, intraparotid lymph node metastasis, advanced non-melanoma skin cancer, head and neck cancer, parotid malignancies

## Abstract

We conducted a retrospective, longitudinal study on a single-center series of patients who underwent parotidectomy in the management of advanced head and neck non-melanoma skin cancer (hnNMSC). The aim of this study was to identify prognostic factors associated with worse outcomes. Forty-one men and nine women were included. The mean age at the time of surgery was 78.9 years. The 5-year overall survival, disease-specific survival, locoregional recurrence-free survival, and distant metastasis-free survival calculated with Kaplan–Meier curves were 39.9%, 56.3%, 58.6%, and 82.1%, respectively. A univariate analysis showed that the status of the margins, facial nerve direct involvement, lymph vascular invasion, and histological grading were associated with worse outcomes (*p* < 0.05). Positive margins were associated with worse disease-specific survival also in a multivariate analysis (*p* = 0.001, HR = 32.02, and CIs 4.338 to 351.3). Because the resection in free margins is the most important prognostic factor, early diagnosis or, in the case of advanced disease, extensive surgical resection with concomitant reconstruction is needed. Adjuvant therapy is indicated in selected cases.

## 1. Introduction

Skin cancers are classified in two major categories: melanoma and non-melanoma skin cancer (NMSC) [1]. They accounted for 330,000 and 1,200,000 new cases worldwide, respectively, in 2022 [2].

Chronic solar ultraviolet radiation is the main etiological factor; as a consequence, most lesions develop from the head and neck in elderly patients [3,4].

Compared to melanoma, NMSC has a lower propensity to distant metastasis and an overall better survival [5].

The WHO reports 82 types of NMSC [1], but most of the tumors are squamous cell carcinoma (SCC), basal cell carcinoma (BCC), and Merkel cell carcinoma (MCC) [1,6].

NMSC usually presents as a small lesion, and simple surgical excision in free margins guarantees a high cure rate, with acceptable cosmetic and functional results.

Approximately 1%-5% of patients with NMSC present with locally advanced disease, with lymph node metastasis, and/or infiltration of contiguous structures [7]. In these patients, survival is significantly reduced [8].

The parotid gland is a well-documented area of lymph node metastasis from head and neck NMSC (hnNMSC) [3,9,10,11,12], particularly in the context of lesions arising from the ear, anterior scalp, temple, and forehead [4].

In 1963, Conley and Arena [13] first described a series of 81 patients who underwent surgical treatment for parotid involvement from non-salivary gland tumors. The survival rate at five years was 12.5%. It was previously considered that non-salivary parotid tumors were rare [14] until O’Brien in 1993 [15] described a series of 242 consecutive parotidectomies, in which metastasis from melanoma and hnNMSC were the main malignant histotypes. Nine years later, in 2002, O’Brien et al. [16] observed that intraparotid metastasis from cutaneous SCC had distinct prognostic behavior. This led to the introduction of a prognostic classification, which distinguished intraparotid lymph node metastasis (PM) from neck lymph node metastasis. Although this classification improves prognostic accuracy, there is still an insufficient correlation with pathological data. The N1S3 classification [17] was thus introduced to improve these aspects. Despite the research in the prognostic value of PM, definitive evidence is still lacking. The AJCC-TNM classification [1], currently the most widely used, does not distinguish between PM and neck metastases, but in its eighth edition, it differentiates between hnNMSC and lesions arising from other areas.

Nowadays, the management of the parotid gland represents the cornerstone of treatment in advanced hnNMSC.

In the case of parotid involvement with delayed diagnosis, patients must undergo extensive resection surgery followed by immediate reconstruction. Adjuvant radiotherapy (RT) can improve locoregional control and survival in these advanced cases [4,18,19].

Despite the improvement of a multidisciplinary approach for advanced hnNMSC, the importance of parotid involvement is still not clear, and many patients experience relapse and die as a consequence of uncontrolled disease [4]. The present study analyzes a single-center series of patients who underwent parotidectomy for advanced hnNMSC. The objective of this study was to describe the experience of a tertiary center in the diagnosis and surgical management of patients with advanced hnNMSC over a ten-year period and to help clinicians tailor the treatment protocol for patients with parotid involvement, together with giving advice to the primary surgeon as to planning rigorous follow-up in the case of resection of hnNMSC.

## 2. Materials and Methods

### 2.1. Study Design, Data Extraction, and Variables

We performed a retrospective, longitudinal analysis approved by the Ethical Committee on Human Clinical Research of our hospital (PG/2016/4730) of all the consecutive patients who underwent parotidectomy for hnNMSC at the Department of Otorhinolaryngology—Head and Neck Surgery of Cagliari State University between January 2011 and May 2022.

Informed consent was obtained from all the subjects involved in this study.

The current study included patients who underwent parotid surgery for PM, those who had undergone surgery for NMSC where the parotid parenchyma was directly infiltrated, and those who had undergone parotidectomy as part of the surgical management of NMSC where the final histology did not demonstrate parotid involvement.

The clinical data were obtained by the review of clinical charts, direct clinical re-examination and imaging of surviving patients, and phone calls to the relatives of deceased patients. All the data were organized with Microsoft Excel (Microsoft; Redmond, WA, USA). The demographic and clinical data included sex, age at surgery, age-adjusted Charlson Comorbidity Index, and physical examination at diagnosis. The histopathological and clinical variables used included the histotypes, site of disease, skin involvement, status of margins, sites of metastasis, extranodal extensions (ENEs), facial nerve (FN) status, perineural invasion (PNI), lymphovascular invasion (LVI), histological grading (G), TNM stage, O’Brien stage, N1S3 stage, and adjuvant treatments. The main outcomes were overall survival (OS), disease-specific survival (DSS), locoregional relapse-free survival (LRFS), and distant metastasis-free survival (DMFS). The outcomes were calculated from the day of surgery to, respectively, the day of death for other causes, day of death disease-related, date of clinical or radiological diagnosis of locoregional relapse, and date of radiological diagnosis of distant metastasis. For censored subjects, the date of the last follow-up was used as the endpoint.

### 2.2. Preoperative Work-Up and Treatment Principles

The preoperative work-up included ultrasonography (US), computer tomography (CT), magnetic resonance imaging (MRI), and histological assessment by fine-needle aspiration cytology (FNAC) and/or incisional biopsy.

The comorbidities were evaluated using the age-adjusted Charlson Comorbidity Index [20].

The parotidectomies were classified according to the European Salivary Gland Society Classification (ESGSC) [21].

The FN dissection was always performed under operative microscopic view or with the 3D exoscope. The systematic intraoperative FN monitoring was always performed using a four-channel free-running electromyography nerve integrity monitor system (NIM 3.0, Medtronic USA, Inc., Jacksonville, FL, USA).

The preoperative and postoperative FN function was evaluated with the House–Brackmann grading system (HB) [22]. A total or partial FN resection was performed in the case of nerve infiltration. In the case of FN resection, intraoperative neurorrhaphy and/or static suspension of the facial palsy was executed to reduce the impact of the FN postoperative deficit.

According to the NCCN Guidelines [23,24], elective neck dissection was always indicated in patients with a clinical diagnosis of parotid or lymph node metastasis.

The patients with cT3 and cT4 cN0 lesions of the auricular or periauricular region underwent prophylactic neck dissection associated with type I-II parotidectomy (superficial parotidectomy).

The surgical defects were classified according to the Rosenthal classification [25]. In the case of extensive resections, the reconstruction was performed with free or pedicled flaps. The extent of surgery, age, comorbidities, and performance status influenced the reconstructive choice.

In the patients with a high risk of postoperative airway impairment (pedicle or free flap reconstruction and bilateral neck dissection), a concomitant elective tracheostomy was performed.

The Clavien–Dindo classification was used for the evaluation of postoperative complications [26].

According to the Royal College of Pathologists guidelines, the margin status was evaluated as follows: complete excision (the tumor is fully excised and surrounded by a margin of normal tissue of at least 1 mm), involved margin (the specimen analyzed shows cancer present at the margin), and close margin (the specimen analyzed shows a fully excised tumor with, however, a ≤1 mm margin of normal tissue at one or more sites of the specimen) [27,28].

SCC, BCC, and MCC were staged according to the 8th edition of the AJCC-TNM [1]. The patients with intraparotid metastasis of SCC were also staged with the O’Brien classification [16] and N1S3 classification [17].

The adjuvant therapy was planned according to the NCCN Guidelines [23,24], following a discussion by a multidisciplinary tumor board. The RT field, dosage, modality, and duration was always planned by the radiation oncologist. Usually, a dose of 54–63 Gray was administered on the P and N, in 30–33 fractions, with conformal radiation therapy or intensity-modulated radiation therapy. Usually, three cycles of Cisplatin were administered by the medical oncologist as the chemotherapy (CHT) regimens.

All the patients were followed-up regularly at our institution for 5 years after surgery to rule out regional or distant relapses. Our protocol consists of a clinical examination every 2 months for the first 2 years, every 3 months for the third year, every 4 months for the fourth year, and every 5 months for the fifth year. Head and neck CT or MRI with contrast medium were scheduled every 6 months for the first 2 years and once every year for the remaining 3 years. Chest CT with contrast medium was performed once a year. Biopsy or cytology were executed in the case of suspected relapse. All the follow-up data were updated until April 2023.

### 2.3. Statistical Analysis

The quantitative variables (age and age-adjusted Charlson Comorbidity Index) were reported as the mean, range and standard deviations (SDs). The qualitative variables were reported as the absolute (N°) and relative frequencies (%). A statistical analysis was performed using GraphPad Prism 9 (San Diego, CA, USA). The Kaplan–Meier curves were calculated for survival outcomes at 5 years (OS, DSS, LRFS, and DMFS). The univariate analysis results were reported as the survival proportion, hazard ratios (HRs), and confidence intervals (CIs). The log-rank, Mantel, and Wilcoxon tests were used to compare the survival curves. Cox proportional hazards regression was used for the multivariate analysis. A *p*-value < 0.05 was considered significant.

## 3. Results

A total of 50 patients were included in our study, which included 41 men (82%) and 9 women (18%) (M/F = 4.5:1). The mean age at the time of surgery was 78.9 years (range: 59–98 years, SD 9.1) (the demographic and clinical features are detailed in Table 1). The mean age-adjusted Charlson Comorbidity Index was 6.7 (range: 4–10, SD 1.2).

A total of 37 patients (74%) had PM at clinical presentation, while 13 patients (26%) underwent resection of the primaries of the periauricular region extended to the parotid gland to obtain free deep margins of resection.

Among the patients with PM, 23 patients (62.2%, 46% of all series) had a clinical history of previous hnNMSC resection without neck or parotid staging performed elsewhere, 13 patients (35.1%, 26% of all series) were treated for primary hnNMSC of the head and neck with concomitant PM, and 1 patient (2.7%, 2% of all series) developed PM 8 months after the resection of SCC of the submandibular skin associated with level I-V neck dissection and adjuvant chemotherapy (CHT-RT) performed at our institution.

The mean time between the treatment of the primary tumor and the diagnosis of PM (available for only 12 cases) was 8.9 months (range: 1–18 months).

The cutaneous primaries are detailed in Table 1.

A preoperative radiological evaluation was obtained for all the patients: 12 patients underwent preoperative work-up with MRI, 30 patients with CT, and 8 patients with MRI and CT. Distant metastases were excluded by chest CT with contrast medium.

The preoperative histology was obtained with incisional biopsy in 26 patients (52%) with a cutaneous lesion. The preoperative diagnosis of malignancy in the patients observed for a parotid lump without a cutaneous lesion was based on the FNAC in 10 patients (20%), and in 14 patients (28%), the diagnosis was based on the clinical history of a recent resection of hnNMSC.

Forty-five patients (90%) had SCC, four patients (8%) MCC, and one patient (2%) BCC.

According to the 8th edition of the AJCC-TNM [1], 24 patients (48%) were cT0, 4 patients (8%) were cT2, 19 patients (38%) were cT3, and 3 patients (6%) were cT4. PM (cP+) was observed in 37 patients; among them, 18 patients (48.6%) were also cN+ and 19 patients (51.4%) were cN0.

The patients underwent 26 right parotidectomies (52%), 22 left parotidectomies (44%), and 2 bilateral parotidectomies (4%). According to Quer et al. [21], we performed type I-II parotidectomy (superficial parotidectomy or exofacial parotidectomy) in 21 cases (40.4%, with partial or total FN resection in 3 cases 5.8%), type I-II-III parotidectomy (superficial parotidectomy extended to the inferior lobe) in 12 cases (23.1%, with partial or total FN resection in 4 cases 7.7%), type I-IV parotidectomy (total parotidectomy with FN preservation) in 3 cases (5.8%), and type I-IV (VII) parotidectomy (total parotidectomy with partial or total FN resection) in 16 cases (30.8%).

In 23 cases (44.2%), the FN was involved by the tumor, but only 8 patients (16%) presented a preoperative FN impairment (HB > 1). The surgical resection was extended to a single branch of the FN in 12 cases (52.2%) and to the main trunk in 11 cases (47.8%), as shown in Table 2. In these cases, intraoperative neurorrhaphy and/or static rehabilitation of the facial palsy was performed to reduce the morbidity of the postoperative FN palsy.

Forty-nine patients (98%) underwent concomitant neck dissection.

A total of 46 patients underwent ipsilateral neck dissection, 2 patients with bilateral parotid involvement underwent bilateral neck dissection (*n* = 4 neck dissection), and 1 patient previously treated with unilateral neck dissection underwent contralateral neck dissection. In total, 51 neck dissections were performed: 28 selective neck dissections (54.9%), 12 functional neck dissections (23.5%), and 11 modified radical neck dissections (21.6%), as shown in Table 2.

One patient (2%) did not undergo neck dissection because of the histology (BCC).

In 28 cases, the surgical resection was extended to the surrounding structures (skin or ear lobe in 21 cases, skin and cortical bone from the mastoid and/or zygomatic process in 6 cases, and skin and mastoid in 1 case). According to the Rosenthal classification [25] of the defects of the lateral region, 12 patients (42.9%) had a type I defect, 12 patients (42.9%) had a type II defect, and 4 patients (14.3%) had a type III defect. The reconstruction was made with a free flap (*n* = 20, 71.4%) or pedicled flap (*n* = 8, 28.6%), as reported in Table 2 and Table 3. The recipient artery for microvascular anastomosis was the facial artery in 17 cases (85%), the superior thyroid artery in 2 cases (10%), and the ascending pharyngeal artery in 1 case (5%). In all the cases, the recipient vein for the microanastomosis was one of the branches of the thyro-lingual-facial trunk. Venous drainage was obtained with a single anastomosis in most cases (*n* = 18, 90%), using the Microvascular Anastomotic Coupler Device (MACD) produced by GEM Synovis MCA [29] in all the cases. The mean time of anastomosis (TA), defined as the operative time of anastomosis of both the arterial and venous sides, was 42.3 min (range 20–60 min). The mean time of ischemia (TI), defined as the period between the detachment of the flap from the donor site and the end of the vascular anastomosis, was 58.8 min (range 30–100 min). We observed one free flap failure (5%) and one pedicle flap failure (12.5%). These two cases were reconstructed with local rotational flaps.

Eighteen patients (36%) had concomitant elective tracheostomy to prevent respiratory distress in the postoperative period. Six patients (12%) had dissection and identification of the trachea, without tracheotomy (pre-tracheostomy), to facilitate airway management in case of emergency. One patient (2%) had an emergency tracheostomy performed for postoperative bleeding. No one patient required a permanent tracheostomy.

Seventeen patients (34%) were admitted in the Intensive Care Unit (ICU) of our hospital after surgery (mean stay in ICU was 1.6 days).

The mean hospitalization time was 14.8 days (range 5–42 days).

According to Clavien–Dindo [26], we considered grade I and grade II as minor complications (*n* = 14, 28%) and grade III and IV as major complications (*n* = 8, 16%), see Table 4.

At definitive histology, PM was confirmed in 37 patients (74%), in 10 patients (20%) the parotid parenchyma was involved by the neoplasm by direct infiltration, and in 3 cases (6%) the parotid was not involved by the tumor, see Table 5.

The primaries in the patients with PM (37 patients, 74% of the whole series) originated from the periauricular region in 15 cases (40.5%), forehead and temple in 7 cases (18.9%), scalp in 5 cases (13.5%), unknown in 3 cases (8.1%), cheek in 2 cases (5.4%), neck in 2 cases (5.4%), periorbital region in 2 cases (5.4%), and nose in 1 patient (2.7%).

The definitive histology showed free margins (R0, >1 mm) in 38 cases (73.1%), close margins (R close, <1 mm) in 8 cases (15.4%), and positive margins (R+) in 6 cases (11.5%). An R close was observed on the T in one patient and on the P in seven patients. Three patients had an R+ on the T not amenable to surgical enlargement: in one case, the lesion was in the frontal region and the deep margin was represented by the frontal bone, while in two patients with parotid infiltration who underwent superficial parotidectomy, the deep margin was represented by the facial nerve. Three patients had an R+ on the N with infiltration of the neck structure not suitable for surgical resection.

According to the 8th edition of the AJCC-TNM [1], 2 patients (4%) were stage II, 20 patients (40%) were stage III, and 28 patients (56%) were stage IV. A total of 24 patients (48%) were pT0, 4 patients (8%) were pT2, 19 patients (38%) were pT3, and 3 patients (6%) were pT4. In total, 9 patients (18%) were pN0, 11 patients (22%) were pN1, 11 patients (22%) were pN2, and 19 patients (38%) were pN3. A total of 41 patients (82%) had nodal metastasis: 4 patients (9.8%) had neck metastasis alone, 15 patients (36.6%) had PM alone, and 22 patients (53.6%) had both neck and PM. Twenty-two patients (53.6%) were ENE+. Nodal metastases involved the parotid in 37 patients (90.2%), with level I in 3 cases (7.3%), level II in 20 cases (48.8%), level III in 11 cases (26.8%), level IV in 6 cases (14.6%), and level V in 6 cases (14.6%). Metastasis at a single level was seen in 13 patients (31.7%), while 28 patients (68.3%) had metastasis at multiple levels. Among the patients with PM from SCC, 19 patients also had neck metastasis (pP+N+). In four patients (21%), neck metastasis was observed only at the definitive histological analysis (occult neck metastasis).

According to the O’Brien classification [16], the patients were staged P1 (*n* = 6, 18.2%), P2 (*n* = 10, 30.3%), P3 (*n* = 17, 51.5%), N0 (*n* = 13, 39.4%), N1 (*n* = 8, 24.2%), and N2 (*n* = 12, 36.4%). According to the N1S3 classification [17], the patients were staged I (*n* = 4, 12.1%), II (*n* = 20, 60.6%), and III (*n* = 9, 27.3%).

After the multidisciplinary evaluation, 30 patients (60%) underwent adjuvant treatment: RT alone in 21 cases, and RT and concomitant CHT in 9 cases. Fifteen patients (30%) could not undergo adjuvant treatment despite indications because of the advanced age (*n* = 8), comorbidities (*n* = 5), and personal reasons (*n* = 2). Adjuvant therapies were not indicated in five patients (10%).

The mean time of follow-up was 2.6 years (range 0.7–10 years).

Sixteen patients (34%) experienced a locoregional relapse. One patient (6.25%) underwent salvage neck dissection and is still alive after 16 months. Fifteen patients (93.7%) were not fit for aggressive salvage surgery or with unresectable disease and underwent palliative treatment (only one patient is still alive). Two patients (4%) experienced locoregional relapse and distant metastasis (one of them underwent immunotherapy and is still alive, while the other patient underwent palliative treatment and died of disease 16 months after the recurrence). Four patients (8%) experienced distant metastasis and died of disease.

The five-years OS, DSS, LRFS, and DMFS of all the series were 39.9%, 56.3%, 58.6%, and 82.1%, respectively.

We did not observe statistically significant differences between the patients with PM (*n* = 37) and the patients with direct parotid infiltration from NMSC (*n* = 10) (5-years DSS: 62.7% versus 40%, *p* = 0.2535; 5-years LRFS: 59.8% versus 54%, *p* = 0.9734; and 5-years DMFS: 81% versus 85.7%, *p* = 0.7762).

Positive margins of resection (R+) were associated with a worse prognosis in the univariate analysis in all the patients of the present series (see Table 6 and Table 7).

FN infiltration, LVI, and G were associated with worse prognosis in the univariate analysis in patients with SCC PM. The O’Brien and N1S3 classifications showed good prognostic value for LRFS. The statistical analyses of the patients with intraparotid metastases of NMSC are detailed in Table 8, Table 9 and Table 10.

The univariate analysis highlighted that the margins’ status, FN infiltration, LVI, and G are prognostic risk factors in patients with parotid metastasis from SCC (see Table 8 and Table 9).

The multivariate analysis of these risk factors showed that only positive margins of resection were associated with a significantly worse DSS (*p* = 0.001), as shown in Table 11.

## 4. Discussion

The majority of NMSCs are small superficial skin lesions, associated with a good prognosis, with a DSS at 5 years greater than 90% [30]. Treatment modalities reported in the literature are surgical excision, cryotherapy, topical pharmacotherapy, laser, and radiotherapy [31]. A small percentage of these patients (5%) develop advanced locoregional disease that requires more extensive surgical procedures and multi-modality treatments.

Patients treated for even early hnNMSC should always undergo close follow-up so as to detect the onset of parotid or neck metastasis. A parotid swelling observed within two years after the resection of a potentially metastatic hnNMSC should be considered as highly suspicious for metastasis as reported in the literature (PM occurs within 10 to 19 months of the treatment of the primary lesion) [32] and as observed in our series (8.9 months, range of 1–18 months).

Risk factors for advanced disease are tumor diameter, depth of invasions, histological grading, recurrent tumor, and immunosuppression [33]; these characteristics are often neglected at primary surgery. Lesions arising from the preauricular region have also been identified as a high risk for locoregional involvement [27].

The parotid is the most frequent site of metastasis from hnNMSC [3,7,9,10,11,12] and can also be involved by direct infiltration.

A diagnostic work-up of advanced hnNMSC is based on imaging, histology, and cytology [34]. MRI is generally considered more accurate in the diagnostic assessment of parotid lesions [35]. In our series, most of the patients were evaluated by MRI (24%), and patients with highly suspicious intraparotid metastasis referred to our institution with a CT performed elsewhere did not undergo further imaging (60%) unless the parotid or temporal areas required a finer assessment of the deep extension (16%).

The histological diagnosis of lesions arising or involving the skin is confirmed by a biopsy (52% of our patients), while the diagnosis of metastasis is routinely made by FNAC (as observed in 10% of our patients). In our series, we did not consider a preoperative histological or cytological assessment necessary in 28% of patients with a clear clinical correlation between the parotid swelling and the resection of the primary (in 13 cases the first treatment was performed elsewhere and in 1 case in our center). As for parotid lymphoma [36], a standardized diagnostic work-up is still lacking for secondary parotid lesions, with ultrasound representing an emerging diagnostic tool. Recently, the administration of gas contrast medium has enhanced the accuracy of ultrasound in differentiating between benign and malignant lesions [37].

Parotidectomy is the gold standard in the case of hnNMSC metastatic to the parotid. SCC is the most common lesion that can metastasize to the parotid lymph nodes. MCC is an aggressive but uncommon lesion originating from neuroendocrine cells of the skin [38] with an incidence of nodal metastasis of 26% [39]. Accordingly, with their incidence, in our series, 89.2% of the PM were from SCC and 8.1% from MCC. BCC rarely involves regional lymph nodes (from 0.003% to 0.1% as reported in the literature) [39]. In our series, we observed only one case (2.7%) of PM from metatypical BCC, which is considered the most aggressive form of BCC in the literature [40].

Parotidectomy is also indicated in the case of close proximity or direct involvement of the gland from the primary lesion. In our series, 13 patients with periauricular NMSC (*n* = 12 SCC and *n* = 1 MCC) underwent wide resection with concomitant parotidectomy. In 10 of these patients (76.9%), the histology showed the gland’s infiltration, while only in 3 cases (23.1%) the parotid was not involved.

Parotidectomies should be extended to the deep lobe of the gland only when it is macroscopically involved, because lymph nodes are rarely observed in the deep lobe [34,41,42,43].

In our series, neck dissection was indicated in all cN+ patients with SCC and MCC.

Because of the potential risk of occult metastasis, all the patients with PM but without clinical node involvement (cP+N0) of our series underwent neck dissection. The risk of occult neck disease observed in the literature in cP+N0 patients is 22.5% [44]. This is similar to the rate of occult neck metastasis observed in our series of patients (21%). As a consequence, unilateral selective neck dissection (levels II-V) is indicated in these patients [45]. Contralateral neck dissection is still not mandatory [23], and in our series, we never observed contralateral metastasis during the follow-up.

Elective neck dissection is also indicated in the case of NMSC directly infiltrating the parotid gland because the advanced T stage (at least T3) is associated with a higher risk of nodal metastasis [46]. In our series, 4 out of 10 patients with parotid infiltration (40%) presented neck metastasis at definitive histology.

Sentinel node biopsy could be a treatment option in NMSC with a risk of occult nodal metastasis higher than 10% [47], but it was never indicated in our series of patients because of the advanced stage of disease (see Table 5).

Another major challenge in patients with hnNMSC with involvement of the parotid region is the management of the FN, because its sacrifice has a negative impact on quality of life. Direct nerve infiltration with preoperative palsy is a clear indication for nerve sacrifice [34], while in patients without preoperative nerve impairment, it can be difficult to predict when a partial or complete sacrifice of the nerve is necessary to achieve an oncologically sound resection [48]. Conservative nerve-sparing approaches have been reported in the literature in the cases of intraoperative evidence of FN involvement with preoperative normal function, leaving microscopic residual disease involving the nerve, followed by timely postoperative RT [49,50]. However, in such cases, postoperative unpredictable events that delay the RT could have a dramatic impact on oncologic outcomes. According to O’Brian et al. [16], our surgical strategy aimed to preserve the FN only when its function was clinically normal preoperatively and macroscopic clearance of the tumor could be achieved at the time of surgery. The dissection of the FN was performed with the aid of an intraoperative microscope or a 3D exoscope [34,43] to improve the intraoperative assessment of the FN neoplastic involvement and to avoid improper FN sacrifices (all the resected branches of the FN showed neoplastic involvement at definitive histology). An intraoperative macroscopic involvement of the nerve was observed in 36.6% of patients with preoperative normal FN function (15 out of 41 patients). In total, 17 patients (45.9%) with parotid metastasis required the sacrifice of the FN (12 patients underwent resection of at least one branch of the nerve and 5 patients underwent total nerve resection), followed by intraoperative neurorrhaphy (in the case of sacrifice of one or a few branches) or static rehabilitation of the facial palsy (in the case of resection of the main trunk). These data are similar to those reported in the literature [19,42,46].

In the case of extended head and neck resections, a reconstruction is generally required. In our series, most of the reconstructions were performed with microvascular free flaps (antero-lateral tight free flap in 70% of cases, forearm free flap in 25% of cases, and rectus abdominis free flap in 5% of cases). A pectoralis major pedicled flap was chosen only in four patients with a high risk for free flap failure (previous head and neck RT, severe chronic kidney disease, and an age-adjusted Charlson Comorbidity Index higher than nine), while the platysma pedicled flap was used in four patients with an age-adjusted Charlson Comorbidity Index higher than eight who underwent minor skin resection. Our success rate of free tissue transfer (95%) is in line with other studies from the literature [25].

The Rosenthal classification [25] showed a good correlation between the surgical defect and the modality of reconstruction in 89.3% of patients (see Table 3). A total of 3 out of 28 patients (10.7%) underwent a different reconstruction from what was indicated by the Rosenthal classification. Two patients with a type I defect (parotidectomy extended to the skin with preservation of the auricle) were reconstructed with an ALT flap because of the extensive resection of the check and neck skin, and one patient with a type III defect (parotidectomy extended to the ear and mastoidectomy) was reconstructed with a radial forearm free flap because of the limited surface of the area of resection.

According to Clavien–Dindo [26], we observed a minor complication rate of 28% and a major complication rate of 16% (see Table 4). The relatively high complication rate of our series is mainly due to the advanced mean age of our patients and the advanced stage of disease and consequent aggressive treatment required, as already reported in the literature [51].

According to the NCCN [23], adjuvant radiotherapy in SCC patients is indicated in the case of PNI, multifocal nerve invasion, recurrent tumor, a maximum diameter >6 cm, close margins when further surgery is not indicated, and desmoplastic or infiltrative tumors in immunosuppressed patients. In patients with MCC, the NCCN Guidelines [24] recommend RT in the case of a maximum diameter >1 cm, LVI, chronic T-cell immunosuppression, HIV, chronic lymphocytic leukemia, solid organ transplant, and a head and neck primary site. The NCCN Guidelines [23] do not recommend adjuvant CHT for most cases of fully resected regional SCC. Adjuvant CHT is used in a clinical trial with advanced locoregional SCC, but the supporting data are limited [23]. There is no indication for adjuvant CHT in fully resected primary MCC [24]. CHT is indicated in the case of recurrent disease [24].

Our 5-year OS (39.9%), DSS (56.3%), and LRFS (58.6%) are similar with the survival outcomes reported in the literature [11,52,53].

We compared the OS, DSS, LRFS, and DMFS of 10 patients with direct parotid infiltration by NMSC with those of 37 patients with PM from NMSC, and we observed similar outcomes.

The staging of SCC with parotid metastasis can be conducted with the AJCC TNM, O’Brien, and N1S3 classifications [1,16,17].

In the 8th edition of the TNM [1], the side, size, number of metastatic lymph nodes, and ENE are considered to differentiate the N1, N2, and N3 categories. According to the literature [11,46,53], in our study, N+ patients experienced a worse but not statistically significant prognosis compared to N0 patients, as shown in Table 7.

There is an emerging interest in the literature about the different prognostic behaviors between parotid (P) and neck (N) metastasis from NMSC [1,23,54].

O’Brien et al. in 2002 [16] described a classification that differentiates the parotid lymph node (P) from the neck lymph node (N). In 2003, Carsten et al. [55] observed a good statistical correlation between the extent of the parotid disease on the basis of the O’Brien classification and survival outcomes in patients with intraparotid metastasis from SCC. In our series, the O’Brien [16] classification showed a better correlation in the prediction of LRFS than the TNM [1] classification (see Table 8).

In 2010, Forest et al. [17] described a new classification (N1S3) in which the number and dimensions of neck and parotid metastases differentiate the N categories: stage I single metastasis measuring ≤3 cm; stage II single metastasis >3 cm or multiple measuring ≤3 cm; and stage III multiple metastases measuring >3 cm. In our series, the N1S3 classification showed a better correlation with DSS and LRFS than the other classifications (see Table 8), as observed by other authors [56].

We did not observe differences in DSS, LRFS, and DMFS between the patients with PM and the patients with neck metastasis from SCC (see Table 8 and Table 9).

According to the literature [57,58], our analysis showed that the margins of resection, FN infiltration, LVI, and grading (G3-G4) were associated with worse outcomes in the patients with PM from SCC (see Table 8 and Table 9).

Free margins of resection were associated with better outcomes than close or positive margins (DSS 65.7% versus 23.6%, *p* = 0.008; LRFS 72.7% versus 12.5%, *p* = 0.005; and DMFS 86.9% versus 52.5%, *p* = 0.01). The patients with close margins (<1 mm) experienced better outcomes than the patients with positive margins (DSS 40% versus 0%, *p* = 0.001; LRFS 20% versus 0%, *p* = 0.002). Our multivariate analysis confirmed that an R+ was associated with a worse DSS (*p* = 0.0011, see Table 11). These data show that radical resection is mandatory to improve outcomes. Unfortunately, the complex anatomy and the cosmetic implications of the head and neck are associated with the high incidence of close and positive margins of resection reported in the literature [27].

Myers et al. [46] reported that FN infiltration was associated with a reduced DSS and Shao et al. [59] reported a reduced OS. In our series, FN infiltration was associated with worse LRFS (80.1% versus 39.1%, *p* = 0.04), especially in the case of resection with close or positive margins, although without statistical significance (LRFS 75% versus 16.7%, *p* = 0.1). These data support the indication of the resection of the FN because a surgical approach aiming to its preservation at the cost of leaving macroscopic neoplasm may worsen the prognosis.

LVI is a well-identified risk factor in patients with head and neck SCC [29,57]. In the univariate analysis, LVI was associated with a worse prognosis (DSS 68.2% versus 30%, *p* = 0.04; DMFS 84.2% versus 53.3%, *p* = 0.04). Other authors observed that LVI is associated with a worse DSS [11,46,52] and, as a consequence, adjuvant CHT could be indicated in these cases.

In the literature, a high-grade tumor (G3-G4) is reported as a negative prognostic factor in cutaneous SCC [57]. In the univariate analysis, we also observed that the high-grade tumor (G3-G4) of the patients with PM from SCC was associated with a poorer outcome (DSS 84.4% versus 37.5%, *p* = 0.04), supporting the indication of adjuvant therapies in the case of poorly differentiated and undifferentiated SCC.

Patients with ENE+ experienced a worse although not statistically significant prognosis, as reported by other authors [11,52,53].

We did not observe a correlation between the size of the PM and the prognosis (see Table 8 and Table 9). We considered the 3 cm cut-off as reported by the TNM classification system [1] and by O’Brien [16] and Forest [17]. Laxague et al. [52] reported a worse DSS in patients with parotid metastasis > 5 cm^3^, but none of our patients had metastasis of this size.

Other authors associated PNI with worse outcomes [11,46], but in our series, we did not find any correlation.

Immunodepression is an important risk and prognostic factor reported in the literature [59,60]. In our cohort of patients, only three patients (6%, *n* = 2 transplant and *n* = 1 chronic hematological malignancies) were considered immunosuppressed according to the NMSC United Kingdom Guideline [60]. In these patients, the univariate analysis was not possible because of the low number of cases. Two patients died of other causes after 1 and 6 months of the treatment. One patient is still alive after 3.6 years despite a second primary SCC of the oropharynx treated with surgery.

Adjuvant RT or CHT can improve locoregional control and survival outcomes in selected patients with PM of NMSC [9]. Some authors [11,53,61,62] observed that multi-modality treatments are associated with better outcomes, but our results did not show benefits from RT and CHT (see Table 10). Probably, the small number of patients and the high comorbidity index (mean Charlson Comorbidity Index was 6.7) of our series could be a relevant bias in the analysis of the effectiveness of adjuvant treatments.

Immunotherapy has been recently introduced in patients with unresectable or metastatic SCC, but further studies are required to evaluate the real effectiveness [63,64].

Regardless of the oncological outcome, extensive surgical resection inevitably results in a reduction in patients’ quality of life. As a consequence, in addition to survival, immunotherapy will also have to compete with surgery in terms of residual quality of life.

Our study shows several limitations. Primarily, it is a monocentric study based on a review of medical charts. The statistical analysis has limited power due to the small sample size. Additionally, most of our patients were observed after the resection of the primary tumor performed in another institution, which means that data about the primary lesion are lacking. Furthermore, our population was associated with a high prevalence of comorbidities and advanced age.

## 5. Conclusions

Our results confirm that the involvement of the parotid gland by metastasis or direct infiltration of NMSC is associated with a decreased prognosis.

Our finding leads to two important conclusions. Firstly, it is imperative to perform a preoperative imaging examination to rule out any early involvement of the parotid or neck lymph nodes in patients diagnosed with hnNMSC. Secondly, a rigorous follow-up program should be planned for patients treated for hnNMSC, even in cases of early stage disease, mostly for lesions originating from the ear, temple, and anterior scalp.

Because the resection in free margins is the most important prognostic factor, early diagnosis, extensive surgical resection, and concomitant reconstruction are required. Subsequent adjuvant therapy is indicated in selected cases.

We found that N1S3 was the more accurate system to predict the prognosis in patients with parotid metastasis from SCC. In these patients, positive margins of resection, FN infiltration, LVI, and high grade (G3–G4) represent negative prognostic factors.

## Figures and Tables

**Table 1:** Demographics and clinical data of patients who underwent parotidectomy for NMSC.

		N (%)
**Age** (mean—range—SD)	78.9 years—59–98 years—9.1	50
**Age-adjusted**		
**Charlson Comorbidity Index**	6.7	
(mean range–SD)	(4–10–1.2)	
**Sex**	Male	41 (82%)
	Female	9 (18%)
	Ratio (M/F)	4.5:1
**Side of parotidectomy**	Right	26 (52%)
	Left	22 (44%)
	Bilateral	2 (4%)
**Preoperative work-up**	CT	30 (60%)
	MRI	12 (24%)
	MRI + CT	8 (16%)
	FNAC	10 (20%)
	Biopsy	26 (52%)
**Sites of cutaneous primaries**	Periauricular region	26 (52%)
	Forehead and temple	7 (14%)
	Scalp	5 (10%)
	Cheek	4 (8%)
	Unknown	3 (6%)
	Periorbital	2 (4%)
	Neck	2 (4%)
	Nose	1 (2%)
Preoperative nerve involvement *	No	29 (55.8%)
	Yes	23 (44.4%)

* Considering 52 procedures.

**Table 2:** Detailed surgical procedure (IGV = internal jugular vein, SCM = sternocleidomastoid muscle, and AN = accessory nerve).

			N (%)
**Type of parotidectomy** (according to ESGSC’s classification)	Parotidectomy I Parotidectomy I-II Parotidectomy I-II-III Parotidectomy I-IV Parotidectomy I-IV (VII)		0 (0%)
			21 (40.4%)
			12 (23.1%)
			3 (5.8%)
			16 (30.8%)
**Facial nerve resection**	Partial		12 (52.2%)
		Cervico-facial	7 (30.4%)
		Temporo-zygomatic	3 (13%)
		Marginalis mandibulae	2 (8.8%)
	Complete		11 (47.8%)
**Neck dissection**	No		1 (2%)
	Yes		49 (98%)
Types of neck dissections *	Partial		28 (54.9%)
		II-V levels	19 (67.9%)
		Ib-V levels	6 (21.4%)
		II-III levels	2 (7.1%)
		I-IV levels	1 (3.6%)
	Functional neck dissection		12 (23.5%)
	Modified radical		11 (21.6%)
	neck dissection		
		I-V levels + IGV	1 (9.1%)
		II-V levels + SCM	1 (9.1%)
		II-V levels + SCM + AN	1 (9.1%)
		I-V levels + SCM + AN	1 (9.1%)
		I-III levels + SCM + AN + IGV	1 (9.1%)
		I-V levels + SCM + AN + IGV	2 (18.2%)
		I-V levels + SCM	4 (36.3%)
**Primary intention closure**			22 (44%)
**Reconstruction**			28 (56%)
	Free flap		20 (71.4%)
		Antero-lateral tight	14 (70%)
		Radial forearm	5 (25%)
		Rectus abdominis	1 (5%)
	Pedicled flap		8 (28.6%)
		Pectoralis major	4 (50%)
		Platysma	4 (50%)

* Considering 51 procedures as indicated in the main text.

**Table 3:** Reconstruction according to Rosenthal’s classification for temporal region defect.

	Pedicled Flap		Free Flap		
Class Defect N (%)	Platysma	Pectoralis Major	Radial Forearm	Antero-Lateral Tight	Rectus Abdominis
**I** 12 (42.9%)	4 (33.3%)	4 (33.3%)	2 (16.7%)	2 (16.7%)	0 (0%)
**II** 12 (42.9%)	0 (0%)	0 (0%)	2 (16.7%)	10 (83.3%)	0 (0%)
**III** 4 (14.3%)	0 (0%)	0 (0%)	1 (25%)	2 (50%)	1 (25%)

**Table 4:** Postoperative complications according to Clavien–Dindo [26].

**Minor complications** (*n* = 14, 28%)	Grade I (*n* = 6, 12%)	Wound infections opened at the bedside (*n* = 4, 66.7%) Medical condition without needs for pharmacological, surgical, endoscopic, or radiological interventions (*n* = 2, 33.3%)
	Grade II (*n* = 8, 16%)	Pharmacological treatment with drugs other than such allowed For grade I complications (*n* = 5, 62.5%) Blood transfusion (*n* = 2, 25%) Total parenteral nutrition (*n* = 1, 12.5%)
**Major complications** (*n* = 8, 16%)	Grade IIIb (*n* = 6, 12%)	Postoperative bleeding (*n* = 4, 57.1%) Free flap failure (*n* = 1, 16.7%) Pedicle flap failure (*n* = 1, 14.3%)
	Grade IVa (*n* = 2, 4%)	Respiratory distress (*n* = 2, 100%)

**Table 5:** Pathological data, adjuvant therapy, and follow-up.

		N (%)
**Histological diagnosis**	SCC	45 (90%)
	BCC	1 (2%)
	MCC	4 (8%)
**Parotid involvement and definitive histology**	Intraparotid metastasis	37 (74%)
	SCC	33 (89.2%)
	MCC	3 (8.1%)
	BCC	1 (2.7%)
	Parotid infiltration	10 (20%)
	SCC	9 (90%)
	MCC	1 (10%)
	Parotid not involved	3 (6%)
	SCC	3 (100%)
**Intraparotid SCC metastasis size**	≤3 cm	19 (57.6%)
	>3 cm	14 (42.4%)
**T stage**	T0	24 (48%)
	pT1	0 (0%)
	pT2	4 (8%)
	pT3	19 (38%)
	pT4	3 (6%)
**N stage**	pN0	9 (18%)
	pN+	41 (82%)
	P-N+	4 (9.8%)
	P+N-	15 (36.6%)
	P+N+	22 (53.6%)
	ENE+	22 (53.6%)
**Stage**	I	0 (0%)
	II	2 (4%)
	III	20 (40%)
	IV	28 (56%)
**Status of margins**	R0	38 (73.1%)
	R close (<1 mm)	8 (15.4%)
	R+	6 (11.5%)
**Adjuvant treatment**	Radiotherapy	21 (42%)
	Radio-chemotherapy	9 (18%)
	No	20 (40%)
**Relapse**	No	28 (56%)
	Yes	22 (44%)
	Locoregional	16 (32%)
	Locoregional + distant metastasis	2 (4%)
	Distant metastasis	4 (8%)

**Table 6:** Univariate analysis of margin status; significative values are highlighted in bold. Levels of significance are reported as * (*p* ≤ 0.05), ** (*p* < 0.01), *** (*p* < 0.001).

		N (%)	5-Year DSS (SE)	*p*-Value	5-Year LRFS (SE)	*p*-Value	5-Year DMFS (SE)	*p*-Value
**All series**	R0	36 (72%)	65.5% (9.1)	0.01 * (Mantel) 0.02 * (Wilcoxon)	72% (8)	0.0051 ** (Mantel) 0.01 * (Wilcoxon)	88.1% (6.7)	0.0278 * (Mantel) 0.0435 * (Wilcoxon)
	R close and R+	14 (28%)	27.9% (15.5)		20.4% (12.7)		59.3% (18.5)	
**All series**	R close	8 (57.1%)	44.4% (22.2)	0.0004 *** (Mantel) 0.001 ** (Wilcoxon)	33.3% (19.2)	0.0007 *** (Mantel) 0.0019 ** **(Wilcoxon)**	66.7% (19.2)	0.0614
	R+	6 (42.9%)	0%		0%		50% (35.4%)	
**All series**	R0	36 (81.8%)	65.5% (9.1)	0.5582	72% (8)	0.2704	88.1% (6.7)	0.0972
	R close	8 (18.2%)	44.4% (22.2)		33.3 (19.2)		66.7% (19.2)	
**All SCC**	R0	33 (73.3%)	65.7% (9.6)	0.0086 ** (Mantel) 0.0117 * (Wilcoxon)	72.7% (8.2)	0.0054 ** (Mantel) 0.0231 * (Wilcoxon)	86.9% (7.3)	0.0199 * (Mantel) 0.0327 * (Wilcoxon)
	R close and R+	12 (26.7%)	23.6% (14.4)		12.5% (11.6)		52.5% (20.4)	
**All SCC**	R close	7 (58.3%)	40% (21.9)	0.0011 ** (Mantel) 0.0025 ** (Wilcoxon)	20% (17.9)	0.0024 ** (Mantel) 0.0059 ** **(Wilcoxon)**	60% (21.9)	0.0833
	R +	5 (41.7%)	0%		0%		50% (35.5)	
**All SCC**	R0	33 (82.5%)	65.7% (9.6)	0.4172	72.7% (8.3)	0.1590	86.9% (7.3)	0.0988
	R close	7 (17.5%)	40 (21.9)		20% (17.9)		60% (21.9)	
**Intraparotid metastasis from SCC**	R0	25 (75.8%)	74.6% (10.1)	0.0120 * (Mantel) 0.0175 * (Wilcoxon)	77.3% (9)	0.0076 ** (Mantel) 0.0273 * **(Wilcoxon)**	89% (7.6)	0.0094 ** (Mantel) 0.0211 * (Wilcoxon)
	R close and R+	8 (24.2%)	28.6% (17.1)		15% (13.8)		41.7% (22.2)	
**Intraparotid metastasis from SCC**	R close	4 (50%)	50% (25)	0.0101 * (Mantel) 0.0143 * (Wilcoxon)	100%	0.0207 * (Mantel) 0.0339 * (Wilcoxon)	50% (25)	0.1573
	R+	4 (50%)	0%		25% (21.7)		50% (35.4)	
**Intraparotid metastasis from SCC**	R0	25 (86.2%)	74.7% (10.1)	0.3681	77.3% (9)	0.1131	88.9% (7.6)	0.0337 * (Mantel) 0.0553 (Wilcoxon)
	R close	4 (13.8%)	50% (25)		25% (21.7)		50% (25)	

**Table 7:** Hazard ratios, *p*-value, and 95% confidence intervals for univariate analysis of margin status; significative values are highlighted in bold.

		DSS			LRFS			DMFS		
		HR	*p*-Value	95% CI	HR	*p*-Value	95% CI	HR	*p*-Value	95% CI
**All series** **(*n* = 50)**	**R close and R+ versus R0**	4.359	**0.01**	1.284 to 14.80	4.885	**0.005**	1.609 to 14.83	11.44	**0.02**	1.305 to 100.2
	**R+ versus R close**	61.92	**0.0004**	6.221 to 616.3	28.66	**0.0007**	4.133 to 198.7	90.2	0.06	0.807 to 10042
	**R close versus R0**	1.544	0.5	0.3608 to 6.606	2.188	0.2	0.5437 to 8.807	8.498	0.09	0.6779 to 106.5
**All SCC** **(*n* = 45)**	**R close and R+ versus R0**	5.5	**0.008**	1.542 to 19.62	5.494	**0.005**	1.656 to 18.22	14.07	**0.01**	1.519 to 130.3
	**R+ versus R close**	40.46	**0.001**	4.398 to 372.3	28.32	**0.002**	3.266 to 245.6	54.6	0.08	0.5907 to 5046
	**R close versus R0**	1.888	0.4	0.04067 to 8.766	2.902	0.1	0.6588 to 12.79	10.61	0.09	0.7839 to 143.6
**Intraparotid metastasis from SCC** **(*n* = 33)**	**R close and R+ versus R0**	7.659	**0.01**	1.564 to 37.51	7.134	**0.007**	1.686 to 30.18	22.95	**0.0094**	2.157 to 244.1
	**R+ versus R close**	25.79	**0.01**	2.170 to 306.4	17.42	**0.02**	1.546 to 196.3	20.09	0.1573	0.3142 to 1284
	**R close versus R0**	2.535	0.3	0.3344 to 19.21	4.513	0.1	0.6998 to 38.11	22.4	**0.03**	1.270 to 395.1

**Table 8:** Univariate analysis for patients with intraparotid metastases from SCC; significative values are highlighted in bold. Levels of significance are reported as * (*p* ≤ 0.05), ** (*p* < 0.01), *** (*p* < 0.001), **** (*p* < 0.0001).

Variables		N (%)	5-Year DSS (SE)	*p* Value	5-Year LRFS (SE)	*p* Value	5-Year DMFS (SE)	*p* Value
**Sex**	Male	28 (84.8%)	60% (10.7)	0.7120	62% (10.1)	0.4527	78.5% (9.9)	0.7695
	Female	5 (15.2%)	75% (21.7)		60% (21.9)		75% (21.7)	
**Age**	≤79 years	18 (54.5%)	63.8% (12)	0.6129	59.5% (11.9)	0.9375	77.9% (11.3)	0.8228
	>79 years	15 (45.5%)	65.3% (14.2)		65.6% (14.5)		83.9% (10.4)	
**Site of disease**	T + N	12 (36.4%)	53.5% (15.5)	0.4671	78.8% (13.4)	0.1340	68.8% (15.7)	0.3230
	N	21 (63.6%)	70% (11.4)		52.1% (11.7)		84.6% (10)	
**Skin involvement**	No skin infiltration	22 (66.7%)	57.1% (16.4)	0.4736	46.8% (16.7)	0.3504	74.1% (16.1)	0.5843
	Skin infiltration	11 (33.3%)	65.5% (11.7)		68.4% (10.8)		80.8% (10.3)	
**Margins**	Free margins	25 (75.8%)	74.7% (10.1)	<0.0001 **** (Mantel) 0.0002 *** (Logrank)	77.3% (8.9)	0.0032 ** (Mantel) 0.0012 ** (Logrank)	89% (7.6)	0.0172 * (Mantel) 0.0045 ** (Logrank)
	Close margins	4 (12.1%)	50% (25)		25% (21.7)		50% (25)	
	Positive margins	4 (12.1%)	0% (0)		0% (0)		50% (35.4)	
**Sites of metastasis**	Superficial lobe	25 (75.8%)	62.1% (10.9)	0.8567	62.4% (10.7)	0.9690	73.2% (10.5)	0.2176
	Superficial and deep lobe	8 (24.2%)	66.7% (19.2)		58.3% (18.6)		100%	
**Sites of nodal** **metastases I**	P+N0	14 (42.4%)	73.8% (13.1)	0.3731	71.8% (14)	0.2579	80.8% (12.6)	0.9178
	P+N+	19 (57.6%)	53% (13.5)		53.3% (12.2)		76% (12.7)	
**Sites of nodal** **metastases II**	P+N0	14 (77.8%)	73.8% (13.1)	0.1350	71.8% (14)	0.0816	80.8% (12.6)	0.4371
	P0N+	4 (22.2%)	25% (21.7)		25% (21.7)		100%	
**Extranodal extensions**	ENE-	14 (42.4%)	72.2% (13.8)	0.4424	70% (14.5)	0.2278	81.5% (12)	0.8970
	ENE+	19 (57.6%)	52.7% (14)		55.4% (11.9)		86.6% (12.8)	
**Facial nerve status**	Spared facial nerve	17 (51.5%)	67.8% (12)	0.5609	80.1% (10.4)	0.0491 * (Mantel) 0.0788 (Log-rang)	79.1% (11.1)	0.9435
	Infiltred facial nerve	16 (48.5%)	57% (14.8)		39.3% (14.1)		77.8% (13.9)	
**PNI**	PNI-	15 (45.5%)	71.8% (11.9)	0.4782	77% (11.8)	0.1711	86.1% (9.1)	0.6374
	PNI+	18 (54.5%)	46.8% (16.9)		47.8% (13.1)		68.1% (15.8)	
**LVI**	LVI-	28 (84.8%)	68.2% (10.1)	0.0612 Mantel 0.0445 * (Wilcoxon)	67% (9.7)	0.0672	84.2% (8.6)	0.0626 Mantel 0.0455 * (Wilcoxon)
	LVI+	5 (15.2%)	30% (23.9%)		30% (23.9)		53.3% (24.8)	
**Grading** (data available only for 26 patients out of 33)	G1-G2	15 (57.7%)	84.4% (10.1)	0.0465 * (Mantel)	69.8% (12.8)	0.2569	90% (9.5)	0.1171
	G3-G4	11 (42.3%)	37.5% (18.9)		40.9% (17.6)		63% (17.7)	
**Intraparotid metastasis diameter**	≤3 cm	19 (57.6%)	66.6% (11.2)	0.02556	70.7% (11.2)	0.2392	88.8% (7.5)	0.3000
	>3 cm	14 (42.4%)	45.7% (21.2)		48.4% (15.1)		60% (18.2)	
**O’Brien (P)**	P1	6 (18.2%)	100%	0.1467	100%	0.0851 (Mantel) 0.0270 * (Logrank)	100%	0.2633
	P2	10 (30.3%)	48% (16.4)		65.6% (16.4)		64% (17.5)	
	P3	17 (51.5%)	57.3% (14.8)		39.9% (14.2)		77.8% (13.9)	
**O’Brien (N)**	N0	13 (39.4%)	81.5% (11.9)	0.3758	80.2% (12.8)	0.0924 (Mantel) 0.0370 * (Logrank)	80.2% (12.8)	0.9947
	N1	8 (24.2%)	53.6% (20.1)		62.5% (21.3)		85.7% (13.2)	
	N2	12 (36.4%)	49.9% (16.4)		41.7% (14.2)		72.9% (16.5)	
**N1S3**	I	4 (12.1%)	100%	0.0395 * (Mantel) 0.0137 * (Logrank)	100%	0.0105 * (Mantel) 0.0045 * (Logrank)	100%	0.3734
	II	20 (60.6%)	72.1% (10.8)		70.3% (11.4)		82.5% (9.3)	
	III	9 (27.3%)	0%		25.4% (15.5)		37.5% (28.6)	
**pN**	N1	5 (15.2%)	80% (17.9)	0.4722	80% (17.9)	0.3240	100%	0.5115
	N2	10 (30.3%)	72.9% (16.5)		66.7% (19.2)		72.9% (16.5)	
	N3	18 (54.5%)	48.5% (15)		52.7 (12.3)		73.3% (14)	

**Table 9:** Hazard ratios, *p*-value, and 95% confidence intervals for univariate analysis of patients with intraparotid metastases from SCC; significative values are highlighted in bold.

Variables	DSS			LRFS			DMFS		
	HR	*p*-Value	95% CI	HR	*p*-Value	95% CI	HR	*p*-Value	95% CI
**Male versus female**	1.4	0.7	0.234 to 8.346	0.4856	0.4	0.07369 to 3.200	0.6943	0.7695	0.06046 to 7.973
**>79 years**	0.7084	0.6129	0.1864 to 2.693	1.050	0.9208	0.3081 to 3.581	0.8098	0.5062	0.1278 to 5.130
**T+N**	1.605	0.4671	0.4485 to 5.741	0.3966	0.1340	0.1184 to 1.329	2.481	0.3230	0.4092 to 15.05
**Skin infiltration**	1.644	0.4736	0.4221 to 6.403	1.827	0.3504	0.5157 to 6.472	1.717	0.5369	0.2475 to 11.92
**Superficial lobe versus superficial and deel lobe**	1.147	0.8567	0.2581 to 5.100	0.9738	0.9690	0.2556 to 3.710	3.691	0.2176	0.4629 to 29.43
**P+N+ versus P+N0**	1.765	0.3731	0.5055 to 6.163	1.991	0.2579	0.6040 to 6.560	1.098	0.9178	0.1847 to 6.532
**P+N0 versus P0N+**	0.2390	0.1350	0.03659 to 1.561	0.1744	0.0816	0.02443 to 1.245	3.715	0.4371	0.1357 to 101.7
**ENE+ verus ENE-**	1.639	0.4424	0.4644 to 5.788	2.078	0.2278	0.6331 to 6.818	1.124	0.8970	0.1909 to 6.620
**Infiltrated facial nerve**	1.458	0.5609	0.4091 to 5.197	3.328	**0.0491**	1.005 to 11.03	0.9374	0.9435	0.1570 to 5.596
**PNI+**	1.572	0.4782	0.4503 to 5.488	2.289	0.1711	0.6992 to 7.494	1.527	0.6374	0.2624 to 8.892
**LVI+**	6.561	**0.0445**	0.9157 to 47.01	6.154	0.0672	0.8794 to 43.07	12.51	**0.0455**	0.8752 to 178.7
**G3-G4** (data available only for 26 patients out of 33)	4.860	**0.0465**	1.025 to 23.05	2.190	0.2569	0.5649 to 8.487	5.076	0.0922	0.6655 to 38.71
**Intraparotid metastasis diameter >3 cm**	1.111	0.8730	0.3066 to 4.024	2.091	0.2392	0.6123 to 7.139	2.610	0.3000	0.4254 to 16.01
**All NMSC adjuvant treatments**	1.656	0.4492	0.4487 to 6.109	1.092	0.8833	0.3371 to 3.538	2.127	0.4624	0.2842 to 15.92
**Only SCC adjuvant treatments**	1.478	0.5506	0.4099 to 5.327	1.248	0.7226	0.3671 to 4.243	2.155	0.4562	0.2860 to 16.23

**Table 10:** Univariate analysis of adjuvant treatment outcomes.

	N (%)	5-Year DSS (SE)	*p*-Value	5-Year LRFS (SE)	*p*-Value	5-Year DMFS (SE)	*p*-Value
**All NMSC**							
**Adjuvant treatments**	30 (66.7%)	61.9% (9.7)	0.4492	61.6% (9.1)	0.8833	84.8% (8.2)	0.4624
**No adjuvant treatments**	15 (33.3%)	52.7% (18.8)		59.9% (17)		83.6% (10.8)	
**Only SCC**							
**Adjuvant treatments**	26 (65%)	60.3% (10.6)	0.5506	59.5% (10)	0.7226	82.3% (9.4)	0.4562
**No adjuvant treatments**	14 (35%)	52.4% (18.8)		65% (17.6)		81.4% (11.9)	

**Table 11:** Multivariate analysis of patients with SCC intraparotid metastasis. # Value not reported by the program. Level of significance is reported as ** (*p* < 0.01).

	DSS		LRFS		DMFS	
	Hazard Ratios (95% CI)	*p*-Value	Hazard Ratios (95% CI)	*p*-Value	Hazard Ratios (95% CI)	*p*-Value
**FN infiltration**	0.7062 (0.07692 to 4.520)	0.7249	4.784 (0.7205 to 94.27)	0.1634	0.1718 (0.002649 to 3.25)	0.3210
**LVI+**	2.869 (0.2859 to 34.72)	0.3683	5.722 (0.3399 to 215.9)	0.2921	12.87 (0.3601 to 1935)	0.2068
**G3-G4**	2.598 (0,5501 to 13,31)	0.2243	0.5120 (0.01400 to 7.879)	0.6813	1.986 (0.1982 to 20.52)	0.5378
**Margins’ status (R+)**	32.02 (4.338 to 351.3)	0.0011 **	#	>0.9999	14.17 (0.5108 to 433.1)	0.0767

## Data Availability

All the available data are already reported in the text. Our excel database with a code for the names of the patients is available under request for scientific purpose.
